# Development and validation of a nomogram for predicting 28-day mortality in critically ill patients with acute gastrointestinal injury: prospective observational study

**DOI:** 10.3389/fnut.2024.1469870

**Published:** 2024-10-10

**Authors:** Youquan Wang, Yanhua Li, Yuhan Zhang, Huimei Wang, Yuting Li, Liying Zhang, Chaoyang Zhang, Meng Gao, Hongxiang Li, Dong Zhang

**Affiliations:** ^1^Department of Critical Care Medicine, The First Hospital of Jilin University, Changchun, China; ^2^Department of Gastroenterology, The First Hospital of Jilin University, Changchun, China

**Keywords:** acute gastrointestinal injury, feeding intolerance, nutritional support intensive care unit, outcome, nomograms

## Abstract

**Objective:**

Developing and validating a clinical prediction nomogram of 28-day mortality in critically ill patients with acute gastrointestinal injury (AGI).

**Methods:**

Firstly, the construction of a clinical prediction model was developed using data obtained from a prospective observational study from May 2023 to April 2024. Then, data from a prospective multicenter observational study conducted in the intensive care units of 12 teaching hospitals in 2014 were utilized to independently and externally validate the clinical prediction model developed in the first part. We first screened the covariates of the development cohort by univariate cox regression, and then carried out cox regression analysis on the development cohort by backward stepwise regression to determine the optimal fitting model. Subsequently, a nomogram was derived from this model.

**Results:**

A total of 1102 and 379 patients, 28-day mortality occurred in 20.3% and 15.8% of patients respectively, were included in the development and validation cohort, respectively. We developed a nomogram in critically ill patients with AGI and the AGI grade, APACHE II score, Mechanical ventilation (MV), Feeding intolerance (FI) and daily calorie intake (DCI) in 72 h, were independent predictors of 28-day mortality, with the OR of the AGI grade was 1.910 (95% *CI*, 1.588–2.298; *P* < 0.001), the *OR* of APACHE II score was 1.099 (95% *CI*, 1.069–1.130; *P* < 0.001), the *OR* of MV was 1.880 (95% *CI*, 1.215–2.911; *P* = 0.005), the *OR* of FI was 3.453 (95% *CI*, 2.414–4.939; *P* < 0.001) and the DCI > 0.7 or < 0.5 of calorie target is associated with increased 28-day mortality, with *OR* of 1.566 (95% *CI*, 1.024–2.395; *P* = 0.039) and 1.769 (95% *CI*, 1.170–2.674; *P* = 0.007), respectively. Independent external validation of the prediction model was performed. This model has good discrimination and calibration. The DCA and CIC also validated the good clinical utility of the nomogram.

**Conclusion:**

The prediction of 28-day mortality can be conveniently facilitated by the nomogram that integrates AGI grade, APACHE II score, MV, FI and DCI in 72 h in critically ill patients with AGI.

## 1 Introduction

The gastrointestinal (GI) tract serves as a vital organ, playing essential roles in digestion, absorption, secretion, and immunity (1). However, the GI tract of critically ill patients is notably fragile, often accompanied by functional disturbances such as dysmotility or malabsorption, alterations in microbial composition, and mucosal injury. Factors including infection, elevated intra-abdominal pressure, and inadequate perfusion also contribute to varying degrees of damage to the GI tract (2). Studies had indicated that over 60% of critically ill patients experience GI symptoms, which are closely associated with poorer clinical outcomes (3, 4). Therefore, the GI function of critically ill patients should be given considerable attention.

In 2012, the ESICM Working Group on Abdominal Problems proposed the concept of Acute GI Injury (AGI) to assess GI dysfunction in critically ill patients as part of the multiple organ dysfunction syndrome (5). This provided a novel approach for diagnosing GI dysfunction and formulating nutritional support strategies in critically ill patients. Studies had showed that the incidence of AGI in critically ill patients is approximately 40%, with a mortality rate as high as 33% in AGI patients (6). Furthermore, there is a positive correlation between AGI grade and mortality in critically ill patients (7–10). Hu et al. indicates that persistent feeding intolerance (FI) during the acute phase of critical illness is an independent risk factor for mortality in patients with AGI (9). Li et al. found in their study that the total calorie intake from enteral nutrition (EN) and parenteral nutrition (PN) within 72 h of intensive care unit (ICU) admission is also correlated with the 28-day mortality in critically ill patients with AGI (7). However, these studies have several limitations. Firstly, there was no independent external validation of the predictive factors for mortality in AGI critically ill patients. This lack of validation undermines the credibility and generalizability of the research. Secondly, the considered predictive factors were not comprehensive. A clinical prediction model that comprehensively considers covariates such as the severity of critical illness, the process of nutritional implementation, and feeding outcomes may exhibit higher predictive performance and generalizability.

Therefore, in view of the above limitations, it is very necessary for us to build a high-quality prediction model to predict the mortality of severe AGI patients, so as to accurately identifying the risk factors for mortality in AGI patients and implementing early intervention along with the development of individualized clinical and nutritional treatment plans may improve clinical outcomes, thus holding significant clinical significance and value.

This study aims to develop a predictive model for the 28-day mortality of critically ill patients with AGI, followed by independent external validation. The objective is to enhance the accuracy of prognostic predictions for critically ill patients with AGI, thereby providing valuable insights for clinicians in devising individualized treatment strategies.

## 2 Materials and methods

### 2.1 Study design

This study comprises two components. Firstly, the construction of a clinical prediction model was conducted using data obtained from a prospective observational study conducted in the department of ICU at the First Hospital of Jilin University from May 2023 to April 2024. Basic information and nutrition-related clinical data of patients with AGI were collected through the electronic medical record system. Patients who met the inclusion criteria and did not fulfill the exclusion criteria were included in the development cohort to establish a clinical prediction model aimed at predicting the 28-day mortality among critically ill patients with AGI. The study titled “Development and Validation of a Nomogram for Predicting 28-day Mortality in Critically ill Patients with Acute Gastrointestinal Injury: Mixed Prospective Single-center and Multi-center Observational Study” obtained approval from the Ethics Committee of the First Hospital of Jilin University on March 16, 2023. The research was registered prospectively at the Chinese Clinical Trial Registry (ChiCTR2300071370) on May 12, 2023, and adhered to the ethical guidelines set forth by the Declaration of Helsinki (1975), the regulations ensuring good scientific practice at the First Hospital of Jilin University, and relevant professional codes of conduct. The study was conducted in accordance with the guidelines outlined in the Consolidated Standards of Reporting Trials.

Subsequently, data from a prospective multicenter observational study conducted in the ICUs of 12 teaching hospitals in 2014 were utilized to independently and externally validate the clinical prediction model developed in the first part (hospitals participating in the study are in the Supplementary material) (7). This validation aimed to assess the generalization ability and reliability of the prediction model.

### 2.2 Study patients

Inclusion criteria: (1) Age ≥ 18 years; (2) Meet the definition of AGI (AGI is malfunctioning of the GI tract in critically ill patients due to their acute illness) proposed by the ESICM Working Group in 2012 (5); (3) Failure of one or more organ systems within 24 h of admission to the ICU (Sequential Organ Failure Assessment (SOFA) score ≥ 2 for any single organ system).

Exclusion criteria: (1) Receiving palliative treatment; (2) Death is expected within 72 h; (3) Oral feeding; (4) Pregnant; (5) Missing clinical data; (6) Loss to follow-up.

### 2.3 Data collection

The study involved the collection of comprehensive clinical data from the participants, including baseline clinical characteristics, AGI grade within 24 h of admission, type of AGI (primary/secondary), Acute Physiology and Chronic Health Evaluation II (APACHE II) score, SOFA score, mNUTRIC score, primary diagnosis, mechanical ventilation (MV), continuous renal replacement therapy (CRRT), time of EN initiation, initial feeding rate of EN, feeding tube route, daily caloric intake from EN and parenteral nutrition (PN), use of vasopressors, sepsis, and the highest lactate level within 24 h. Additionally, FI and 28-day mortality were also recorded for analysis.

### 2.4 Assessment of critical illness scores, AGI grade and FI

Critical illness scores, namely the APACHE II score, SOFA score, and mNUTRIC score, were collected in this study. These scores were assigned based on the patient’s worst-case scenario within 24 h of admission to the ICU. Initially, the patient’s clinician conducts an initial evaluation, after which a dedicated researcher (Yuhan Zhang) reviews these scores based on the patient’s clinical data and negotiates in case of errors or differences of opinion.

AGI grading involves the evaluation of both clinical and gastrointestinal symptoms within 24 h of ICU admission by members of the clinical medical team. This assessment is combined with the patient’s internal abdominal pressure to make a comprehensive evaluation.

For the FI assessment, the diagnostic criteria (FI is indicating intolerance of enteral feeding for whatever clinical reason) proposed by ESICM in 2012 are applied (5). The FI evaluation process is conducted independently by two clinicians (Meng Gao and Chao Yang) who were not involved in any data analysis. Their individual assessment results are then integrated by a third clinician (Youquan Wang). In cases where differences arise in the assessment, all three clinicians discuss them, and the final decision is made by the third clinician.

Once all the above evaluation results are determined, they are recorded into the electronic database by full-time scientific researchers and cannot be changed.

### 2.5 Endpoint

The primary endpoint was the 28-day mortality in severely ill patients with AGI. Regular follow-up and timely recording in electronic database.

### 2.6 Sample size consideration

The determination of the sample size was based on the calculation methods advocated by Harrell et al. (11) and Peduzzi et al. (12), which recommend a minimum of 20 outcome events for each predictor variable in a multivariate regression model. During the development of our model, we considered approximately 3–4 critical clinical factors as well as 2–3 scores.

To precisely forecast the occurrence of the 28-day mortality as the outcome event, a minimum of 180 patients experiencing this event was deemed necessary (3 × 3 × 20). This guaranteed a sufficient number of patients experiencing the outcome event in relation to the predictor variables, thereby enabling more trustworthy predictions of 28-day mortality among critically ill patients with AGI.

### 2.7 Statistical analysis

Continuous variables were assessed for normal distribution using the Shapiro-Wilk test. Skewed distributions were analyzed using the Wilcoxon-Mann-Whitney U-test, with results reported as the median and interquartile range. Categorical variables were summarized using frequency (percentage) and compared using either Pearson’s chi-squared test or Fisher’s exact test, as appropriate. Variables with significance at the 0.1 level in univariate analyses were further considered. Collinearity among all covariates was evaluated using Spearman correlation and the Belsley collinearity test.

In order to develop a predictive nomogram indicating the probability of 28-day mortality in critically ill patients with AGI, we initially conducted a multivariate cox regression analysis using a backward stepwise method. This analysis aimed to identify the reduced model within the development cohort. The covariates considered in the analysis included APACHE II score, AGI grade, MV, sepsis, FI, and daily EN + PN calorie intake in 72 h. We derived estimated odds ratios (OR) and 95% confidence intervals (CI). Subsequently, a nomogram was developed based on the refined model, encompassing the identified predictive variables. Each predictor in the nomogram was allotted points by intersecting a vertical line from the respective factor with the point axis. The cumulative sum of points from all predictors was then computed to obtain the total points. By intersecting a vertical line from the total point axis with the 28-day mortality risk axis, one could estimate the probability of 28-day mortality.

Discrimination was evaluated by determining the area under the curve (AUROC) derived from standard receiver operating characteristic (ROC) curves. To evaluate the classification accuracy of the two models, AUROC was compared using the nonparametric approach of DeLong et al. (13). Additionally, various performance metrics such as cutoff, accuracy, sensitivity, specificity, positive and negative likelihood ratios (PLR and NLR), and positive and negative predictive values (PPV and NPV) were calculated for both the development and validation models. The best-fit model and nomogram underwent validation and calibration using bootstrapping techniques (14). The bootstrap method was applied with 1000 resamples, and the resulting bootstrap-corrected AUROC along with a 95% CI were reported. The calibration plots of the nomogram were evaluated using the Hosmer-Lemeshow test. The discrimination and calibration of the nomogram model were validated in an independent external validation cohort. Additionally, decision curve analysis (DCA) and clinical impact curve (CIC) were used to evaluate the clinical utility of the nomogram.

The statistical analysis was carried out using SPSS for Mac version 26 (SPSS Inc, Chicago, IL, USA) and R v4.3.1 (R Foundation for Statistical Computing, Vienna, Austria) using RStudio v1.0.136 (RStudio Inc, Boston, MA, USA).

## 3 Results

### 3.1 Clinical and demographic data for development cohort

**FIGURE 1:**
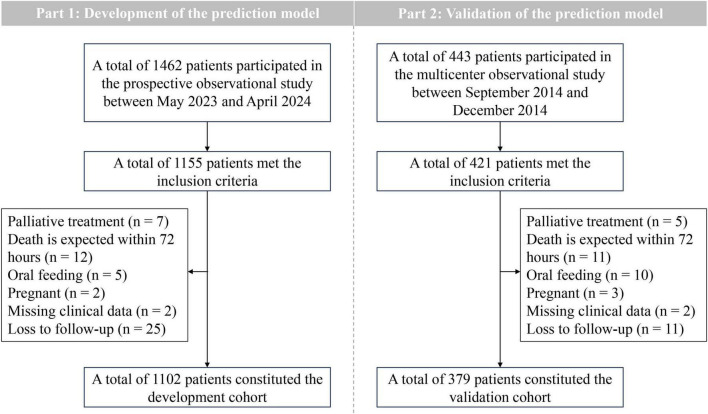
Flow chart of inclusion and exclusion.

During the study period, 1462 patients were admitted, of whom 1155 met the inclusion criteria. Among these, 7 patients received palliative care, 12 patients were predicted to die within 72 h, 5 patients received oral feeding, 2 patients were pregnant, 2 patients missing clinical data and 25 patients were loss to follow-up. Consequently, 1102 patients comprised the development cohort, with a 20.3% incidence of the outcome event (28-day mortality) (Table 1 and Figure 1).

**TABLE 1:** Clinical and demographic data for development and validation cohort.

Variables	Development cohort				Validation cohort			
	Total (*n* = 1102)	Death (*n* = 224)	Survival (*n* = 878)	*P*-value	Total (*n* = 379)	Death (*n* = 60)	Survival (*n* = 319)	*P-*value
Age, mean (SD), y	62.4 (15.4)	64.1 (15.5)	61.9 (15.4)	0.056	61.2 (18.7)	63.9 (21.3)	60.7 (18.1)	0.219
Gender, No. (%)				0.785				0.753
Male	668 (60.6)	134 (59.8)	534 (60.8)		253 (66.8)	39 (65.0)	214 (67.1)	
Female	434 (39.4)	90 (40.2)	344 (39.2)		126 (33.2)	21 (35.0)	105 (32.9)	
BMI, mean (SD)	23.1 (3.9)	22.8 (3.9)	23.2 (3.8)	0.101	23.6 (2.8)	23.2 (2.9)	23.7 (2.7)	0.260
Primary diagnosis, No. (%)				0.681				0.349
Neurologic	223 (20.2)	46 (20.5)	177 (20.2)		73 (19.3)	12 (20.0)	61 (19.1)	
Cardiovascular	66 (6.0)	10 (4.5)	56 (6.4)		24 (6.3)	5 (8.3)	19 (6.0)	
Respiratory	473 (42.9)	102 (45.5)	371 (42.3)		170 (44.9)	20 (33.3)	150 (47.0)	
Multi trauma	75 (6.8)	12 (5.4)	63 (7.2)		25 (6.6)	5 (8.3)	20 (6.3)	
Others	265 (24.0)	54 (24.1)	211 (24.0)		89 (23.5)	18 (30.0)	69 (21.6)	
**Critical care related score ^a^**								
APACHE II, median (IQR)	16.0 (12.0–20.0)	19.5 (14.0–23.8)	15.0 (11.0–19.0)	<0.001	16.0 (12.0–22.0)	22.0 (18.0–26.0)	15.0 (11.0–20.0)	<0.001
SOFA, median (IQR)	7.0 (5.0–9.0)	8.0 (6.0–10.0)	6.0 (4.0–8.0)	<0.001	6.0 (3.0–8.0)	8.0 (5.0–12.0)	5.0 (3.0–8.0)	<0.001
AGI grade, No. (%)				< 0.001				<0.001
I	524 (47.5)	62 (27.7)	462 (52.6)		141 (37.2)	16 (26.7)	125 (39.2)	
II	370 (33.6)	76 (33.9)	294 (33.5)		173 (45.6)	23 (38.3)	150 (47.0)	
III	148 (13.4)	48 (21.4)	100 (11.4)		48 (12.7)	12 (20.0)	36 (11.3)	
IV	60 (5.4)	38 (17.0)	22 (25.1)		17 (4.9)	9 (15.0)	8 (2.5)	
mNUTRIC, median (IQR)	4.0 (3.0–5.0)	4.0 (3.0–5.8)	4.0 (3.0–5.0)	0.262	5.0 (3.0–6.0)	6.5 (4.0–7.0)	5.0 (3.0–6.0)	<0.001
**Underlying disease, No. (%)**								
Hypertension	587 (53.3)	116 (51.8)	471 (53.6)	0.619	201 (53.0)	33 (55.0)	168 (52.7)	0.739
Diabetes	323 (29.3)	67 (30.0)	256 (29.2)	0.825	114 (30.1)	19 (31.7)	95 (29.8)	0.770
Primary AGI, No. (%)	179 (16.2)	34 (15.2)	145 (16.5)	0.628	169 (44.6)	25 (41.7)	144 (45.1)	0.619
MV ^b^, No. (%)	839 (76.1)	192 (85.7)	647 (73.7)	<0.001	258 (68.1)	55 (91.7)	203 (63.6)	<0.001
CRRT ^b^, No. (%)	253 (23.0)	55 (24.6)	198 (22.6)	0.525	50 (13.2)	7 (11.7)	43 (13.5)	0.704
Sepsis ^a^, No. (%)	415 (37.7)	97 (43.3)	318 (36.2)	0.051	160 (42.2)	30 (50.0)	130 (40.8)	0.183
Vasopressors ^a^, No. (%)	525 (47.6)	126 (56.3)	399 (45.4)	0.004	185 (48.8)	38 (63.3)	147 (46.1)	0.014
Maximum lactic acid ^a^, median (IQR), mmol/L	1.9 (1.3–2.8)	2.0 (1.5–3.3)	1.8 (1.3–2.8)	0.014	2.1 (1.4–3.1)	2.6 (1.9–4.7)	2.0 (1.4–2.8)	0.001
EN start time, mean (SD), h	15 (2–44)		16 (2–46)	0.045	48 (24–72)	48 (30–96)	48 (24–72)	0.006
EN initial rate, mean (SD), ml/h	20 (20–30)	20 (15–27.5)	20 (20–30)	<0.001	20 (20–20)	20 (12.5–20)	20 (20–20)	<0.001
FI ^b^, No. (%)	516 (46.8)	170 (75.9)	346 (39.4)	<0.001	161 (42.5)	34 (56.7)	127 (39.8)	0.015
Tube feeding route, No. (%)				0.211				0.703
Prepyloric	1016 (92.2)	211 (94.2)	805 (91.7)		329 (86.8)	53 (88.3)	276 (86.5)	
Postpyloric	86 (7.8)	13 (5.8)	73 (8.3)		50 (13.2)	7 (11.7)	43 (13.5)	
Daily EN calorie intake in 72 h, median (IQR), kcal/kg/day	7.2 (0–17.5)	0.9 (0–10.7)	8.0 (0–18.9)	<0.001	1.1 (0–8.2)	0.0 (0–8.0)	1.4 (0–8.2)	0.556
Daily EN + PN calorie intake in 72 h, No. (%)				<0.001				0.072
< 0.5 of calorie target ^c^	364 (33.0)	96 (42.9)	268 (30.5)		134 (35.4)	29 (48.3)	105 (32.9)	
0.5–0.7 of calorie target ^c^	369 (33.5)	53 (26.7)	316 (36.0)		94 (24.8)	12 (20.0)	82 (25.7)	
>0.7 of calorie target ^c^	369 (33.5)	75 (33.5)	294 (33.5)		151 (39.8)	19 (31.7)	132 (41.4)	

BMI, Body mass index; APACHE II, Acute physiology and chronic health evaluation II; SOFA, Sequential organ failure assessment; AGI, Acute gastrointestinal injury; EN, Enteral nutrition; MV, Mechanical ventilation; CRRT, Continuous renal replacement therapy; FI, Feeding intolerance; IQR, interquartile range. Data presented as mean (standard deviation), median (IQR) or *n* (%).

^a^Indicated within 24 h of ICU admission;

^b^Within 7 days of ICU admission;

^c^The calorie target in this study was 25 kcal/kg/day.

### 3.2 Clinical and demographic data for validation cohort

The dataset from a multicenter, prospective, observational study conducted in 2014 served as the validation cohort for this study. Among the 443 patients enrolled in the study, 379 were included in the final dataset for independent external validation of the predictive model (Table 1 and Figure 1).

### 3.3 Development of the prediction model

Variables such as age, gender, body mass index and tube feeding route were removed from the multivariate cox regression analysis as they did not show statistical significance (*P* > 0.1). Furthermore, the SOFA score was excluded due to collinearity with the APACHE II score; The EN formulas, EN initial rate and daily EN calorie intake in 72 h were excluded from consideration due to collinearity with the AGI grade; Vasopressors and maximum lactic acid within 24 h of ICU admission were excluded due to their collinearity with the sepsis. After conducting the cox regression analysis (correlation analysis of covariates in multifactor cox regression is in the Supplementary material), the independent predictors identified were AGI grade, APACHE II score, FI, MV and daily calorie intake (DCI) in 72 h (Table 2).

**TABLE 2:** Multivariate cox regression analysis of predictors for 28-day mortality in the development cohort.

Influencing factors	*OR* (95% *CI*)	*P*-value
AGI grade	1.910 (1.588–2.298)	<0.001
APACHE II score	1.099 (1.069–1.130)	<0.001
FI	3.453 (2.414–4.939)	<0.001
MV	1.880 (1.215–2.911)	0.005
Daily EN + PN calorie intake in 72 h		0.021
0.5–0.7 of calorie target	1.00 (referent)	
>0.7 of calorie target	1.566 (1.024–2.395)	0.039
<0.5 of calorie target	1.769 (1.170–2.674)	0.007

OR, Odds ratio; CI, Confidence Interval; AGI, Acute gastrointestinal injury; APACHE II, Acute physiology and chronic health evaluation II; FI, Feeding intolerance; MV, Mechanical ventilation.

As the AGI grade elevated (*OR*, 1.910; 95% *CI*, 1.588–2.298; *P* < 0.001) or the APACHE II score increased (*OR*, 1.099; 95% *CI*, 1.069–1.130; *P* < 0.001), the probability of 28-day mortality also increased. FI (*OR*, 3.453; 95% *CI*, 2.414–4.939; *P* < 0.001) and MV (*OR*, 1.880; 95% *CI*, 1.215–2.911; *P* = 0.005) were also positively associated with 28-day mortality. Moreover, DCI in 72 h > 0.7 (*OR*, 1.566; 95% *CI*, 1.024–2.395; *P* = 0.039) or < 0.5 (*OR*, 1.769; 95% *CI*, 1.170–2.674; *P* = 0.007) of target calorie was associated with higher 28-day mortality.

**FIGURE 2:**
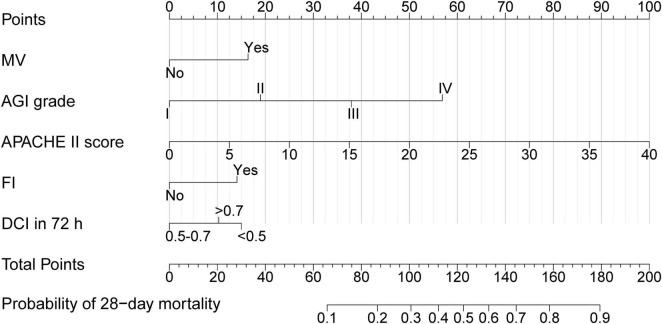
Nomogram of 28-day mortality in AGI patients. MV, Mechanical ventilation; AGI, Acute gastrointestinal injury; APACHE II, Acute physiology and chronic health evaluation II; FI, Feeding intolerance; DCI in 72 h, Daily EN + PN calorie intake in 72 h/target calorie.

The nomogram, which incorporated these predictors, was developed and presented as shown (Figure 2). To obtain the nomogram-predicted probability, the patient’s AGI grade, APACHE II score, FI, MV and DCI in 72 h should be mapped onto the axes of the nomogram predictive factors. A vertical line is drawn on the axes to identify the score for each variable value. By summing up the scores for all variables and locating the corresponding total on the total point line, the individual probability of FI occurrence can be assessed. As an example, an AGI grade II MV patient with the APACHE II score of 15, and FI occurs within 7 days in ICU and DCI in 72 h/target calorie = 0.6. The points corresponding to the nomogram predictor axis were: MV (32 points), AGI (37 points), APACHE (75 points), FI (28 points) and DCI in 72 h (0 points). Add these points together and the total is 172 (32 + 37 + 75 + 28 + 0). According to the nomogram, the patient had 86% risk of 28-day mortality.

### 3.4 Validation of the prediction model

**FIGURE 3:**
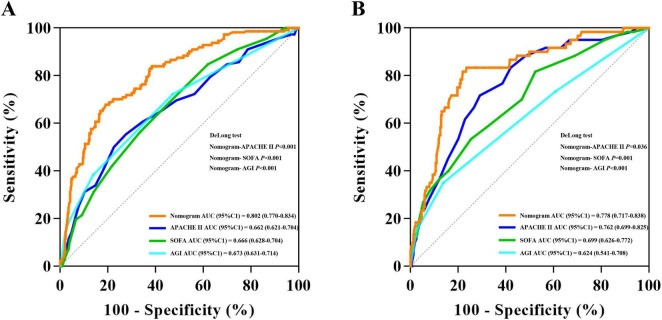
Receiver operating characteristic curve analyses of predictors for 28-day mortality in the development cohort **(A)** and validation cohort **(B)**.

ROC analysis was conducted on the predictors of 28-day mortality in both the development cohort and validation cohort. The AUC (95% CI) were 0.802 (0.770–0.834) and 0.778 (0.717–0.838), respectively, with no significant difference observed (DeLong test, *P* = 0.603). In addition to the nomogram, we performed ROC analyses of APACHE II, SOFA, and AGI grade against 28-day mortality, the nomogram has a higher AUC in the development cohort than APACHE II, SOFA, and AGI grade (DeLong test, *P* < 0.001, *P* < 0.001, *P* < 0.001, respectively). The same results were observed in the validation cohort (DeLong test, *P* = 0.036, *P* = 0.001, *P* < 0.001, respectively) (Figure 3). The 95% CI for the PLR and NLR of the validated model were estimated to be 1.927 (1.668–2.226) and 0.188 (0.087–0.404), respectively. Furthermore, the 95% CI for the sensitivity and specificity of the nomogram were calculated to be 0.900 (0.824–0.976) and 0.533 (0.478–0.588), respectively (Table 3).

**TABLE 3:** Detective characteristics of the development and validation cohort.

Cohort	Cutoff	AUC (95%CI)	Accuracy (95%CI)	Sensitivity (95%CI)	Specificity (95%CI)	PLR (95%CI, %)	NLR (95%CI, %)	PPV (95%CI, %)	NPV (95%CI, %)
Development	0.258	0.802 (0.770–0.834)	0.782 (0.782–0.783)	0.880 (0.844–0.916)	0.679 (0.740–0.809)	3.546 (3.013–4.175)	0.397 (0.328–0.482)	0.475 (0.420–0.530)	0.908 (0.888–0.928)
Validation	0.126	0.778 (0.717–0.838)	0.591 (0.590–0.592)	0.900 (0.824–0.976)	0.533 (0.478–0.588)	1.927 (1.668–2.226)	0.188 (0.087–0.404)	0.266 (0.205–0.327)	0.966 (0.939–0.993)

AUC, area under the receiver operating characteristic curve; CI, confidence interval; PLR, positive likelihood ratio; NLR, negative likelihood ratio; NPV, negative predictive value; PPV, positive predictive value.

**FIGURE 4:**
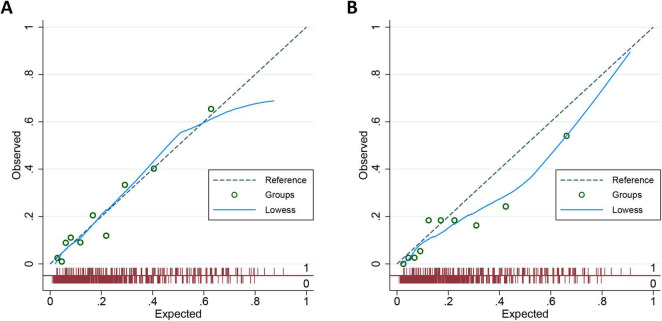
Calibration plot for nomogram in the development cohort **(A)** and validation cohort **(B)**.

To further evaluate the calibration performance of the nomogram model, we plotted a calibration diagram for both the development and validation cohorts (Figure 4). Notably, both curves display slight linearity, indicating excellent calibration performance of the model. To thoroughly assess the calibration of our prediction model, we conducted regression analysis on both the development and validation cohorts, calculating the slope and intercept of the calibration curves. For the development dataset, the slope is 0.9956, which is close to 1, indicating that the model is well-calibrated for this dataset. The intercept is 0.0009 and is not statistically significant (*P* = 0.956), suggesting minimal systematic bias in the model. For the validation dataset, the slope is 0.7720, which is below 1, indicating that the model slightly underestimates in this dataset. The intercept is −0.0035 and is not statistically significant (*P* = 0.890), suggesting a similarly small level of systematic bias. Overall, the prediction model demonstrates good calibration.

### 3.5 Clinical use

**FIGURE 5:**
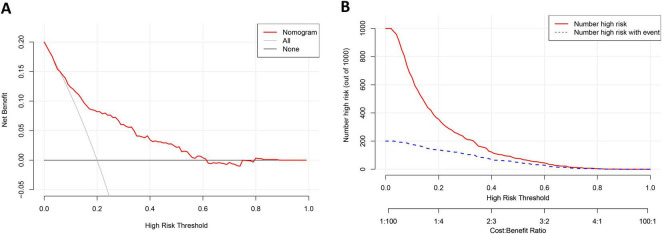
The results of the Decision curve analysis **(A)** and the Clinical impact curve **(B)** of the nomogram for predicting 28-day mortality of AGI patients in the training cohort.

The decision curve analysis of the nomogram of 28-day mortality is shown in Figure 5A. The Y-axis represents the net benefit, while the X-axis represents the threshold probability of 28-day mortality that the clinician considers likely. The light gray curve (All) indicates that the clinician believes that all patients have an outcome event, and the dark gray curve (None) indicates that the clinician believes that all patients have no outcome events. The net benefit is computed by deducting the proportion of patients identified as false positives from the proportion of patients identified as true positives. These values are then weighted, taking into consideration the varying consequences of withholding treatment compared to the negative outcomes associated with unnecessary treatment.

The decision curve shows that if the threshold probability of 28-day mortality is 6–61%, covering the clinically acceptable range (the incidence of 28-day mortality is about 20%), the use of a nomogram has a greater net benefit. For instance, if the threshold probability is 0.2, the net benefit of applying the nomogram model is 0.082. The nomogram model was a more accurate predictor of 28-day mortality in critically ill patients with AGI than the 28-day occurrence of all or none of the outcome events.

The clinical effectiveness of the nomogram was validated through the CIC. The red curve (the number of individuals at high risk) indicates the number of persons who are classified as positive (high risk) by the prediction model at each threshold probability; the blue curve (the number of individuals at high risk with outcomes) is the number of true positives at each threshold probability. The count of individuals identified as high risk (the 28-day mortality cases predicted by the nomogram) closely corresponded to the count of individuals classified as high risk with actual outcomes (the true-28-day mortality cases), particularly when the threshold probability exceeded 40% (Figure 5B).

## 4 Discussion

This study demonstrates that AGI grade, APACHE II score, MV, FI and DCI in 72 h are independent predictive factors for the 28-day mortality in critically ill patients with AGI. Furthermore, a nomogram model with high discrimination, calibration, and clinical use has been developed in this study to predict the probability of 28-day mortality in critically ill patients with AGI. In addition, this study collated and analyzed the risk factors affecting the mortality of AGI patients, and for the first time constructed a column graph that can predict the 28-day mortality of severely ill AGI patients. Although the specificity of the prediction model is slightly lower, it has high sensitivity and meets the practical needs of clinical practice.

In this study, a positive correlation was observed between AGI grade and the 28-day mortality among severe AGI patients, which aligns with our expectations and is consistent with findings from numerous prior investigations in the field (7–10, 15). However, the AGI grade is subject to some subjective limitations. In recent years, Annika et al. proposed a GI dysfunction score (GIDS) aiming to address this issue (16). While GIDS has demonstrated promising performance, it has not successfully integrated serum biomarkers into the final model and still relies on clinical symptoms for assessing GI function, with validation limited to a few studies (17). Therefore, at present, AGI grade retains high utility in the assessment of GI function and prognosis.

In critical care settings, the APACHE II score serves as a reliable measure for assessing the severity of critically ill patients (18, 19). Undoubtedly, whether for AGI patients or any other critically ill individuals, the APACHE II score is among the primary predictive factors considered when estimating mortality. Within the Nomogram, it is clear that this score holds considerable weight in the predictive model. However, due to collinearity concerns, we excluded the SOFA score, as there is significant overlap in the scoring criteria between these two assessments.

In our study, we also found that MV serves as a predictive factor for the 28-day mortality among critically ill AGI patients. The requirement for MV indicates the presence of respiratory failure, potentially compounded by acute respiratory distress syndrome (ARDS), and also increases the likelihood of hospital-acquired infections, particularly ventilator-associated pneumonia (20, 21). Furthermore, these factors may mutually exacerbate, accelerating lung injury and organ failure progression, ultimately leading to prolonged MV duration, extended ICU stays, and the occurrence of ICU-acquired weakness (22, 23).

Due to varying degrees of GI injury among critically ill patients, FI frequently occurs during EN implementation. Numerous studies have indicated an association between FI and higher mortality (9, 24–26), underscoring the importance of monitoring GI symptoms and implementing appropriate interventions for groups at higher risk of FI to potentially improve clinical outcomes for critically ill patients (27). The established predictive model provides a valuable reference for rational intervention strategies. This study included critically ill patients with AGI grades I-IV, and it was easy to find that there was a certain correlation between AGI grade and FI. However, FI was ultimately incorporated into the final predictive model because AGI grade reflects the initial GI status of patients, while FI to some extent reflects the failure of clinical nutrition practices. Therefore, these two predictive factors are considered independent of each other. The role of FI as a predictive factor for the 28-day mortality in AGI patients is not difficult to comprehend, as the nutritional management process for AGI patients is equally crucial. Improper feeding practices may exacerbate GI dysfunction, leading to poorer clinical outcomes even in patients initially classified with lower AGI grades.

This study also found a correlation between the degree of DCI in 72 h and the 28-day mortality in critically ill patients with AGI. It is evident that early full-dose EN or PN can be detrimental (28, 29). This is because the acute phase of critical illness generates substantial amounts of endogenous calories, resulting in lower exogenous energy needs (30). Full-dose feeding can lead to metabolic overload, mitochondrial damage, and inhibition of autophagy, all of which are harmful to critically ill patients (31, 32). This study found that both excessively high and excessively low DCI in 72 h (> 0.7 or < 0.5 of calorie target) increase the 28-day mortality in patients with AGI. The ESPEN guidelines recommend providing low-calorie feeding (not exceeding 70 % of estimated energy expenditure) during the first 72 h of critical illness, as overfeeding has been associated with prolonged hospital stays, extended duration of mechanical ventilation, and increased infection rates. Additionally, studies have shown that low intake ( < 50 %) is associated with poorer clinical outcomes, potentially leading to severe energy deficit, increased muscle loss, and heightened risk of infectious complications (33, 34). For critically ill patients, a phased approach to feeding, transitioning from limited to progressive to open feeding, may be more suitable for AGI patients (32). This study further validates the correlation between DCI in 72 h and the prognosis of critically ill patients. While studies have suggested an association between protein intake and outcomes in critically ill patients (35, 36), our study did not incorporate protein intake of critically ill patients into the predictive model construction. This is because protein intake in critically ill patients is analogous to calorie intake, where early excessive or inadequate intake may lead to overfeeding or malnutrition, with a gradual progression of feeding potentially being more advantageous. Additionally, besides EN and PN, blood products and medications also contribute to protein intake, making the calculation of protein intake more complex and thus increasing the difficulty of nomogram calculation, thereby reducing clinical usability. Therefore, we ultimately opted for calorie intake as an indicator to determine whether early overfeeding or underfeeding exists.

The predictive model developed in this study demonstrates good discrimination and calibration. DCA was further conducted to evaluate the clinical use of the predictive model, revealing that the nomogram yields greater net benefit when the threshold probability of 28-day mortality ranges from 6% to 61%. Additionally, CIC validation was performed to assess the clinical efficacy of the predictive model. The results indicate a close correspondence between the number of individuals classified as high risk when the threshold probability of outcome events exceeds 40% and the number of individuals classified as high risk who actually experience the outcome events. In summary, our findings from multiple perspectives validate the performance and clinical value of the nomogram. The predictive ability and clinical use of the nomogram derived from this study are both excellent.

This study possesses several strengths. Firstly, it establishes, for the first time, a predictive model for 28-day mortality among critically ill patients with AGI, potentially providing valuable insights for clinical decision-making in this population. Secondly, both the development and validation cohorts are derived from independent and prospective observational studies, with the validation cohort sourced from ICU across 12 teaching hospitals in China, thus enhancing the generalizability and robustness of the nomogram developed in this study. Thirdly, the predictors included in the predictive model include both the initial state of the patient and the course of clinical treatment, as the course of clinical treatment (such as FI and DCI in 72 h) is also critical to the outcome of AGI patients.

However, this study has several limitations. Firstly, although the developed and validated cohorts of patients had similar APACHE II scores, there was a significant difference in the proportion of primary AGI cases between the development and validation sets (16.2% vs. 44.6%). Additionally, there are some differences in SOFA and mNUTRIC scores between the two cohorts, and ROC analysis reveals variations in cutoff and accuracy. These differences reflect the heterogeneity among patient populations in the development and validation cohorts. However, this is the best external validation dataset that we are currently able to obtain with accurate AGI grade, despite being a prospective multicenter study conducted nearly a decade ago. Further external validation with additional datasets is needed for a more comprehensive assessment of the prediction model. Secondly, while the predictive model demonstrated strong sensitivity, its specificity was poor. The outcome of nomogram was the 28-day mortality among critically ill AGI patients. For clinicians, it is crucial to accurately identify all individuals at risk of mortality, even if it means some healthy individuals are falsely flagged as having a risk of death. Therefore, sacrificing sensitivity to improve specificity is deemed unacceptable. Nonetheless, it is undeniable that specificity remains one of the limitations of the predictive model. Thirdly, we utilized weight-based prediction equations to calculate calorie targets for the majority of patients, which are known to inaccurately estimate energy expenditure (EE).

## 5 Conclusion

The prediction of 28-day mortality can be conveniently facilitated by the nomogram that integrates AGI grade, APACHE II score, MV, FI and DCI in 72 h in critically ill patients with AGI.

## Data Availability

The raw data supporting the conclusions of this article will be made available by the authors, without undue reservation.
