# Supplementary material for: Development and validation of a nomogram for predicting 28-day mortality in critically ill patients with acute gastrointestinal injury: prospective observational study
Source: Front Nutr. 2024 Oct 10;11:1469870. doi: 10.3389/fnut.2024.1469870 (PMC11499162; doi:10.3389/fnut.2024.1469870)
Supplement: Supplementary file 1 [file Image_1.pdf]

Supplementary document:

Hospitals Participating in the Study

Zhongda Hospital of Southeast University, Nanjing, **East** China

Nanjing General Hospital of Nanjing military Command, Nanjing, **East** China

Zhejiang hospital, Hangzhou, **East** China

First Affiliated Hospital of Sun Yat-sen University, Guangzhou, **South** China

Xiangya Hospital of General south University, Changsha, **Central** China

Zhongnan Hospital of Wuhan University, Wuhan, **Central** China

West China Hospital of Sichuan University, Chengdu, **Southwest** China

First Affiliated Hospital of Xi'an Jiaotong University, Xi’ an, **Northwest** China

First Hospital of Jilin University, Changchun, **Northeast** China

First Hospital of China Medical University, **Northeast** Shenyang, China

First Affiliated Hospital of Dalian Medical University， Dalian, **Northeast** China

Second Affiliated Hospital of Harbin Medical University, Harbin, **Northeast** China

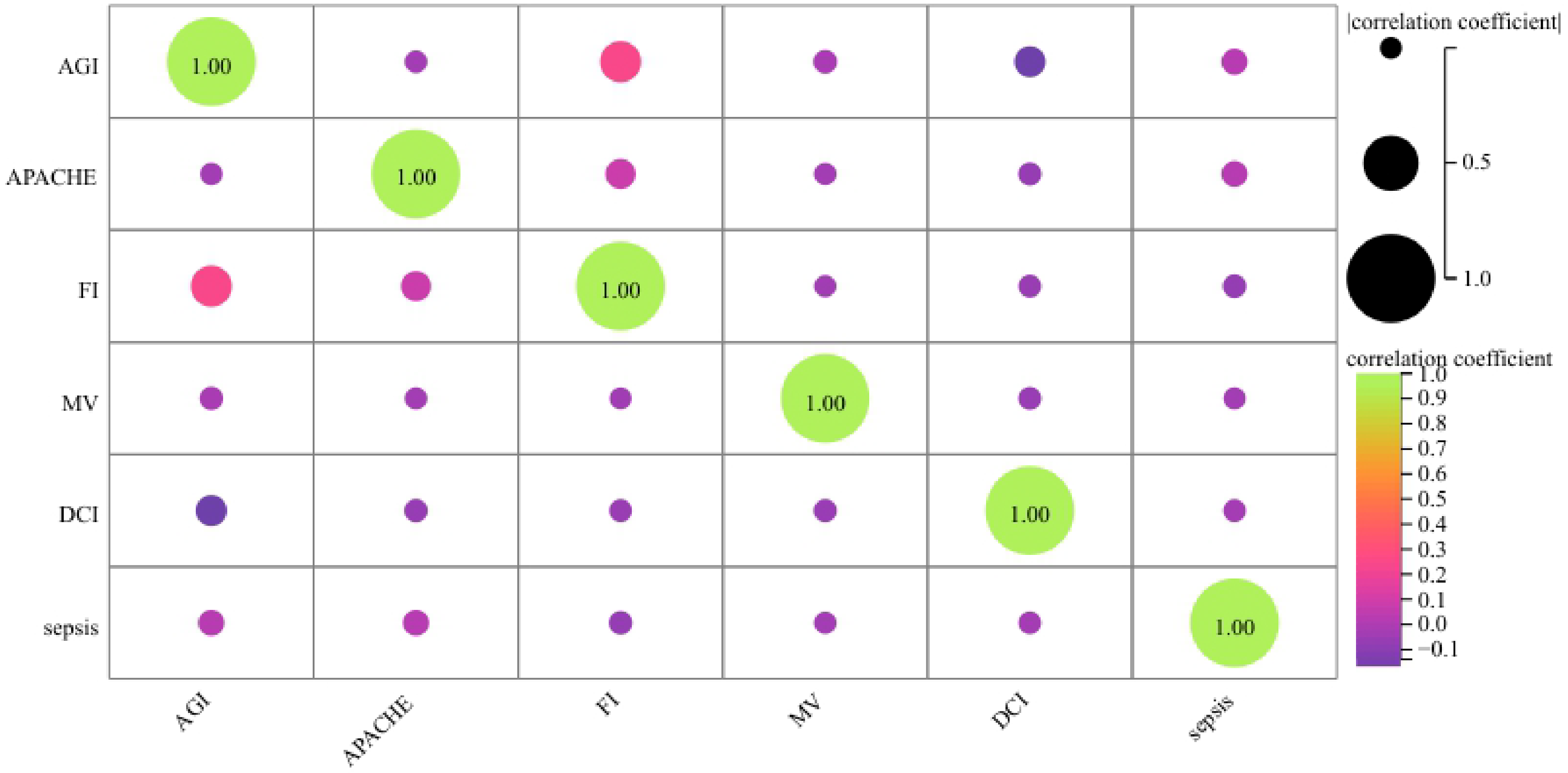

Fig. S1. Correlation analysis of covariates in multifactor logistic regression.
